# Chicago Medical College Commencement

**Published:** 1873-03-15

**Authors:** 


### Medical Examiner.

*A Semi-Monthly Journal of Medical Sciences.*

EDITED BY

N. S. DAVIS, M. D., AND F. H. DAVIS, M. D.

Chicago, March 15th, 1873.

### EDITORIAL.

### CHICAGO MEDICAL COLLEGE COMMENCEMENT.

The public Commencement exercises in this department of the Northwestern University, took place in the College hall, on the 12th and 13th inst.

At 2 o'clock P. M., on the 12th, the programme was commenced with an inaugural thesis, read by John Lewis, on the means for promoting medical science, and especially of obtaining more accurate knowledge concerning the causes and nature of epidemic diseases. It was well written, and indicated much careful thought in arranging plans for investigation.

The candidates for graduation were then subjected to an oral examination, by the Secretary and Dean of the Faculty, for half an hour, on the departments of surgery and practical medicine.

Another thesis was then read by W. F. Wiard, on the nature and objects of medical science. The thesis was well written, abounding in just thoughts, elegantly expressed, and was read in good style.

The oral examination of the class was then resumed for another half-hour, and the exercises were closed by the reading of a thesis by Daniel Lord, on the influence of the study and practice of medicine in developing the whole man. The theme was an interesting one, and was rendered doubly so by the very able manner in which it was discussed by the writer.

On Thursday, at 10 o'clock A. M., the Alumni Association held its annual meeting. There was a good attendance, and the President, Dr. Lyman Ware, delivered an interesting address. After the transaction of the necessary miscellaneous business, the following officers were chosen for the ensuing year: President, Thomas S. Bond, M.D.; first Vice President, R. W. Bronser; second Vice President, D. A. K. Steele, M.D.; Secretary, S. A. McWilliams, M.D., of Chicago.

At 3 o'clock, P. M., the large lecture-room was crowded with an audience of gentlemen and ladies, to witness the conferring of the Degrees. The President of the University, C. H. Fowler, D.D., commenced the exercises by prayer; the Secretary distributed the certificates of progress to the under-graduates, and the certificates for hospital service to those who had actually served as assistants in the Surgical Department of the hospital; and the Dean of the Medical Faculty awarded the prize for the best inaugural thesis to F. J. Huse, of Evanston, and that for the second-best to E. E. Loomis, of Janesville, Wisconsin. The President then conferred the degree of Doctor of Medicine upon forty-one candidates, accompanying the same by a brief, but very appropriate charge. This was responded to, in behalf of the class, by Thomas Killough, in an address, excellent both in the sentiments expressed and in the manner of its delivery. Its only fault was in being a little too long. The more formal valedictory address was then delivered by Prof. Jones, who was listened to with great pleasure and interest.

Throughout the services, at suitable intervals, the audience were delighted with good music, by the Plymouth choir, under the direction of I. V. Flagler. After the benediction, the audience were left to visit the museum, and other parts of the College.

Thus ended the thirteenth collegiate year of the institution. The spring and summer term, inaugurating the fourteenth collegiate year, will commence on the first day of April next, and continue until the commencement of the summer vacation, on the 27th of June. The career of the College thus far has been one of steady, natural growth, highly satisfactory to its friends.

